# Telehealth perceptions and associated factors among older adults with chronic conditions in Saudi Arabia: a comparative study of users and non-users

**DOI:** 10.3389/fpubh.2025.1542974

**Published:** 2025-03-12

**Authors:** Abdulkarim M. Meraya, Amani Khardali, Sarfaraz Ahmad, Ahmed Z. Hoshaibari, Atheer A. Alameer, Raed Y. Jaafari, Hilal A. Thaibah, Moaddey Alfarhan, Mamoon H. Syed

**Affiliations:** Department of Clinical Practice, Faculty of Pharmacy, Jazan University, Jazan, Saudi Arabia

**Keywords:** telehealth, technology acceptance model, perceived ease of use, older adults, chronic conditions

## Abstract

**Background:**

Telehealth is a promising approach for healthcare delivery that uses telecommunication technologies to enhance accessibility by providing remote health services. This study aimed to identify psychosocial factors that best predict behavioral intention to use telehealth/telemedicine, as mediated by attitude toward use, among older adults with chronic conditions in the Jazan region of Saudi Arabia, and to compare users and non-users.

**Methods:**

Participants were aged 60 years or above, residing in Jazan, and presenting with at least one chronic condition. This study used a quantitative research approach with a cross-sectional questionnaire survey. We stratified all analyses by telehealth use status (users or non-users).

**Results:**

Our study sample comprised 466 participants aged 60 or above with at least one chronic condition. The most prevalent condition was diabetes (29%), followed by arthritis (23%). Among the telehealth users (n = 256), a high percentage (42%) reported that telehealth was better than in-person appointments. Among telehealth users, we found that perceived usefulness [(users: β = 0.501; *p* < 0.001) (non-users: β = 0.441; *p* < 0.001)] and facilitating conditions [(users: β = 0.768; *p* = 0.001) (non-users: β = 0.807; *p* < 0.001)] positively affected attitudes to using telehealth. Telehealth users and non-users reported dislike or fear of the service and unavailability of appointments as the most important barriers to using telehealth services.

**Conclusion:**

The results of this study are important for designing new telehealth applications, especially for older adults in Saudi Arabia. Designers and developers must understand that the attributes and capabilities of telehealth applications should be simple and user-friendly to encourage older adults to increase their intention to use telehealth.

## 1 Introduction

Telehealth is a potential approach to a healthcare delivery model that uses telecommunication technologies to enhance healthcare accessibility by providing remote health services to individuals with inadequate access to such services (1). It is the remote delivery and support of health-related services by the adoption of telecommunication and electronic information technology (2). Additionally, it could assist in making healthcare services more accessible and cheaper and maintaining continuity of care (3, 4). Analogously, virtual healthcare is a segment of telehealth involving the use of communication tools such as audio, video, and text messages to deliver care virtually rather than in person (5). The dependency and financial reliance of older adults will surge as the global population increases if older adults are not supported with the assistance they require to perform their daily activities independently (6). Research indicates that older adults' quality of life (QoL) may be significantly improved through the use of information and communication technology (ICT). This improvement could stem from increased connectivity with family, enhanced personal independence, and improved societal engagement. (6). Due to the rapid increase in the population of older adults, financial support and health maintenance have been identified as their two major concerns, and many of these individuals require specialized critical care due to age-related illness and chronic conditions (7). One-sixth of the global population is expected to be aged 60 years or older by 2030. The population of individuals aged 60 years and older is expected to increase from 1 billion in 2020 to 1.4 billion, and then to 2.1 billion by 2050, effectively doubling this age group (8). The demographic composition of the Kingdom of Saudi Arabia indicates that older adults comprise approximately 2.7% of the total population, whereas in Jazan Province, this proportion is slightly higher at 3.6% (9). As individuals grow older, they frequently encounter a range of health issues including diabetes, chronic obstructive pulmonary disease, depression, dementia, and osteoarthritis (8). A considerable proportion of the population of older adults has at least one chronic disorder requiring frequent intensive care and management (10). Self-care is a proactive means of maintaining health by observing symptoms, pursuing therapy, and assessing the effectiveness of the treatment. For more effective management, older adults should be aware of their condition and have the ability and desire to determine goals, overcome complications, and monitor therapeutic outcomes (11). For older adults residing alone, the effective application of information technology along with digital health technology (DHT) appeared most significant in mitigating health expenses and encouraging self-care management (12). Incorporating these technologies in healthcare could alleviate and improve the overall health of older adults at their residences while encouraging their autonomy through telehealth (13).

Remote patient care is facilitated through telehealth, which combines diverse communication technologies and the digital transmission of health data (14). It is not a new concept; however, its application has become common during the previous decade. Notwithstanding the various difficulties, older adults utilize telehealth services when necessary (15). Despite the demonstrated efficacy of telehealth, these technologies are frequently developed without sufficient consideration for their ease of use by patients and caregivers (8). Indeed, most of the telehealth devices in current use were created and implemented regardless of patient opinions about device usability. Usability is the degree to which a patient or caregiver believes a device is user-friendly or simple to use. Telehealth use can be facilitated by evaluating usability, which provides assistance concerning the desired psychological or physical consequences of technology on its users, expediting the practice of telehealth (16). Facilitators are features in either the older patient or the device that help obtain the desired outcome, and barriers are features that hinder its use for the patient (17). With advances in technology and digital healthcare, healthcare accessibility has shifted from a physician-centric model to a patient-oriented approach that enables remote monitoring (18). Due to social restrictions and distancing enforced by the COVID-19 pandemic, DHT has been effectively used to provide safe access to healthcare services and medical facilities (19, 20). The COVID-19 pandemic has revolutionized approaches to developing medical services and underscored the importance of remote health monitoring. Digital healthcare has become the most effective alternative to delivering healthcare to older adults, while reducing exposure to COVID-19 and overload resulting from persistent hospital visits (21). Numerous types of systems have been established to provide healthcare and services to older adults, including wearable devices, mobile health, telemedicine, and artificial intelligence. COVID-19′s impact and risks were substantially mitigated by DHT, which proved instrumental in empowering older adults to track symptoms and manage their wellbeing (22, 23). The use of mobile-health applications during the COVID-19 pandemic displayed progressive effects on the healthcare process and augmented the remote monitoring of healthcare (24).

Despite remarkable advances in DHT, older adults face challenges and barriers due to physical disabilities such as poor vision, cognitive problems, a lack of technological skills, and a lack of perceived ability and time. The digital and e-health literacy of older adults can influence their capacity to use ICT (25). Regardless of advances in healthcare technology, it is essential to assess how older adults perceive this new technology, how they feel about it, and their intention to use it. It is also crucial to identify their barriers to adopting DHT. Older adults' willingness and perspectives are also important when adopting and employing technology-based monitoring systems (26). This study primarily sought to identify which psychosocial factors best predict behavioral intention to use telehealth/telemedicine, as mediated by attitude toward use, among a sample of older adults with chronic conditions in the Jazan region of Saudi Arabia, and to conduct comparisons between users and non-users of telehealth/telemedicine.

## 2 Methods

### 2.1 Participants

The study participants comprised of older adults residing in the southwestern province of Jazan, Saudi Arabia. Individuals were included in this study if they were aged 60 or above and had at least one chronic condition. In order to ensure independent completion of the questionnaire, subjective judgment was used by the data-collectors to determine if there were cognitive impairment/limitations. An evaluation of cognitive functioning was not conducted via a validated scale and self- or proxy-reports (such as by a family member) of cognitive health/decline were not solicited. Prior to participation in the study, trained data collectors verbally evaluated the cognitive functions of the potential participants. By engaging in informal communication, they assessed their language skills and ability to maintain focus and coherence, followed by an evaluation of their orientation, which included inquiries about the present date, location, and recent events. This method helped build a connection with the participants in addition to identifying any communication challenges and verifying preparedness for answering the questionnaire.

### 2.2 Sampling and recruitment

We employed purposive and snowball sampling methods in order to obtain a representative sample. Recruitment of data collectors was done initially by advertising PharmD students at Jazan University's College of Pharmacy, resulting in 34 volunteers. Training was provided to these volunteers on effective communication, techniques to assess cognitive function, and ethical practices for data collection. Potential participants were sought from public places, community centers, and healthcare facilities in the region. Informed consent was obtained before the questionnaire was administered. At the end, participants were requested to suggest other individuals who were eligible for participation in the study. The recruitment process continued through this process until our desired sample size was achieved.

### 2.3 Data collection

A cross-sectional (quantitative) questionnaire was used to evaluate the proposed model. The data collection period was from early June 2023 to mid of mid-September 2023. Both paper-based and online questionnaires were used to collect data. Qualtrics XM was used as the online survey platform. Additionally, the responses obtained through paper-based questionnaires were transferred to Qualtrics XM for analysis and storage. Assistance was provided to participants by data collectors when needed, and clarifications were offered without influencing their responses, thereby ensuring the integrity of the data. In cases where participants had difficulty reading or writing, a proxy respondent, typically a family member, was allowed to assist, given the participant's presence and consent. Completing the questionnaire took ~15–20 min.

### 2.4 Conceptual model

**Figure 1:**
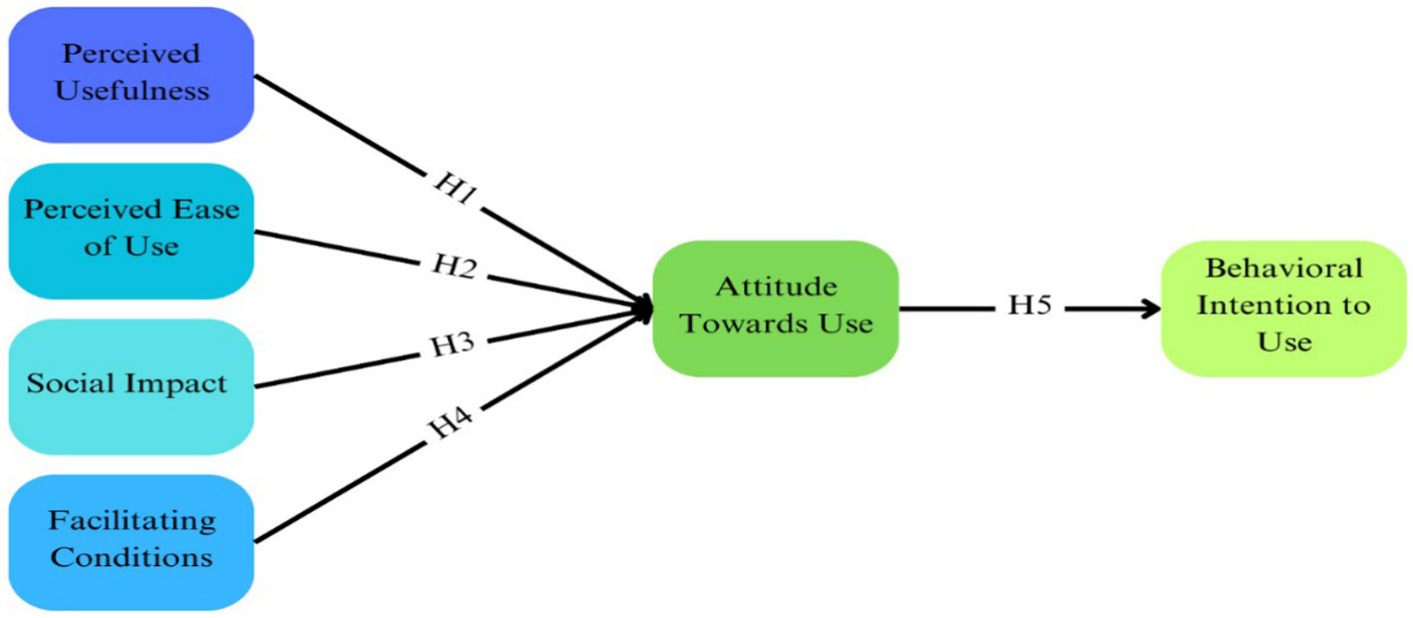
Structural path—technology acceptance model (12).

The technology acceptance model (TAM) is a well-established and widely recognized conceptual model to study the factors influencing individuals' intentions to use telehealth (12, 27–34). The TAM is particularly suited to the older population because it accounts for the perceived usefulness (PU) and perceived ease of use (PEOU) of technology—critical factors for older adults who may be less familiar with digital platforms. Moreover, the TAM has been successfully applied in previous studies involving older adults, demonstrating its effectiveness in understanding technology acceptance in this demographic (12). As shown in Figure 1, the TAM posits that PU, PEOU, social impact, and facilitating conditions affect the attitude toward the use of a particular technology, which affects the behavioral intention to use that technology. PU indicates the extent to which older adults perceive that telehealth can augment their healthcare access, convenience, and quality. PEOU indicates the degree to which older adults perceive telehealth as user-friendly, intuitive, and easy to navigate. Additionally, social influence encompasses the impact of family, relatives, friends, and healthcare professionals in endorsing and encouraging the use of telehealth, while facilitating conditions include the availability of necessary resources, infrastructure, and technical support to facilitate telehealth adoption. Attitude toward telehealth (ATT) was characterized as positive or negative feelings about using a particular technology. Behavioral intention (BI) concerned the use of the technology influenced by one's attitude. BI refers to the extent to which an individual has consciously developed plans to perform or refrain from a specific behavior in future and indicates that a person's BI toward using technology has a positive influence on their actual use of technology. By employing the TAM framework, researchers can study and apprehend the various factors influencing individuals' intentions to use telehealth, enabling the development of effective strategies to enhance telehealth acceptance and use. Hence, we hypothesize that attitude toward using telehealth would mediate the relationship between behavioral intention to use telehealth and perceived usefulness, perceived ease of use, social impact, and facilitating conditions.

### 2.5 Measures

The survey instrument contained the following.

#### 2.5.1 Demographics

The variables included were sex (male or female), age in years, occupation before retirement (office worker, professional, self-employed, blue-collar, dependent, or other), education level (illiterate, high school or less, diploma, bachelor, or more), health insurance (yes or no), chronic conditions (diabetes mellitus, hypertension, heart disease, gastrointestinal complications, dyslipidemia, arthritis, mental illnesses, or other conditions).

#### 2.5.2 Telehealth experience

To distinguish between users and non-users of telehealth services, we asked the question, “Have you had a telemedicine/telehealth appointment (appointment with your health provider by video or phone instead of an in-person appointment)?” We also asked the users, “How did your telemedicine/telehealth visit compare to a traditional in-person medical visit?” with a response scale (better than, just as good as, worse than a traditional visit, and not sure).

#### 2.5.3 TAM variables

The TAM constructs were measured using scales adapted from previous studies that demonstrated good reliability and validity in similar contexts (12, 27–34).

- The PU was evaluated using a 3-item scale: (i) Healthcare using telehealth will help me manage my health. (ii) I believe that using telehealth will make my daily life safer. (iii) Using telehealth will improve my quality of life.
- PEOU also was measured using a 3-item scale: (i) I think using telehealth would be simple. (ii) Learning to use telehealth applications will be easy. (iii) Telehealth-healthcare will be convenient to use.
- Social impact was measured with the following items: (i) Family will approve of my use of telehealth. (ii) Acquaintances will recommend that I use telehealth. (iii) Acquaintances will approve of me using telehealth.
- Facilitating conditions were measured with the following items: (i) I will know how to use telehealth. (ii) If I encounter difficulties using your telehealth, I think someone will be able to help. (iii) I have sufficient resources to use telehealth.
- ATT was measured using a 3-item scale, which included the following statements: (i) Using telehealth will have a positive impact on my life. (ii) Using telehealth will benefit my health. (iii) I have positive thoughts about telehealth.
- BI to use was measured with the following items: (i) I would use telehealth if given the opportunity. (ii) I will use telehealth for my healthcare. (iii) I will use telehealth to change my life for the better.

Participants responded to these questions using a Likert-type scale ranging from “strongly disagree” to “strongly agree.” The instrument was reviewed by experts to ensure content validity. A pilot study with 30 participants was conducted to assess the clarity and reliability of the scales. Cronbach's alpha coefficients for the constructs were >0.70, indicating acceptable internal consistency.

#### 2.5.4 Facilitators to using telehealth

Prior to description of the specific facilitators used in our study, it is crucial to distinguish between facilitating conditions and facilitators to using telehealth. Facilitating conditions assess existing support and resources, including users' perceived ability to use telehealth, access to assistance, and resource availability. In contrast, facilitators to using telehealth address design features and improvements to enhance telehealth acceptance, especially for older users, such as simplified interfaces, guidance systems, and age-appropriate visuals. While facilitating conditions evaluate current support for telehealth use, facilitators to using telehealth explore enhancements for wider adoption. This distinction helps assess present readiness for telehealth and strategies for improving accessibility and acceptance. In this study, facilitators to using telehealth were measured using a 3-item scale, which included the following statements: (i) Devices that use fewer buttons could be a factor that aids in the acceptance of telehealth. (ii) Providing visual and audio guidance could be factors that aid in the acceptance of telehealth. (iii) User-friendly images appropriate for older adults could be factors that aid in the acceptance of telehealth. Participants responded to these questions using a Likert-type scale ranging from “strongly disagree” to “strongly agree.” These items specifically address design features and improvements that could enhance telehealth acceptance, particularly for older users, aligning with the concept of facilitators to using telehealth as described earlier.

#### 2.5.5 Barriers to using telehealth

Participants were asked for their agreement or disagreement on a five-point Likert scale to determine whether the following were barriers to accessing telehealth: (i) The reason that I could not access a telehealth service is that I do not have internet. (ii) The reason that I could not access a telehealth service was dislike or fear of the service. (iii) The reason that I could not access a telehealth service was that an appointment was not available when required. (iv) The reason that I could not access a telehealth service was that it was not available from my general practitioner or other health professional.

### 2.6 Instrument development and validation

The questionnaire was developed based on earlier studies that employed the TAM (12, 27–34) and adapted to the cultural context of the older population in Saudi Arabia. Prior validation studies have shown these scales to be reliable and valid in assessing technology acceptance. Adjustments were made to language and content to ensure relevance and comprehension. The pilot testing helped refine the questionnaire for clarity and cultural appropriateness.

### 2.7 Ethical consideration

This study was approved by the Institutional Review Board and Ethics Committee at Jazan University, Saudi Arabia (reference number: REC44/10/653). The study was performed in accordance with the principles of the Declaration of Helsinki. Each participant provided an informed consent prior to participation. Those who participated in the online survey through Qualtrics XM were provided with detailed information about purpose and procedures of the study and were required to review the consent form and indicate their agreement before proceeding with the questionnaire. The voluntary nature of participation, confidentiality, and anonymity of the responses were emphasized. Participants were also informed of their right to withdraw at any time by simply exiting the survey, without any consequences.

For participants completing the paper-based survey, data collectors elucidated study's purpose and procedures before obtaining signed informed consent. Participants were assured of confidentiality and anonymity of their responses and were notified that their participation was voluntary and that they exit the survey at any time with no consequences. In cases where participants had reading difficulties, consent forms were read aloud, and verbal consent was documented with the participant's approval.

### 2.8 Statistical analysis

Descriptive statistics were generated to summarize participant characteristics. Frequencies and percentages were derived for categorical variables and presented in tables and graphs. Continuous variables were summarized using means and standard deviations. Chi-square tests were performed to examine differences in demographic categorical variables between users and non-users. To evaluate differences in continuous demographic variables between the two groups, *t*-tests were employed. For every scale employed in the present study, an exploratory factor was determined through principal component extraction analysis. For assessing the data suitability for factor analysis, we employed Bartlett's test whereas sampling adequacy was checked by Kaiser-Meyer-Olkin test (35). To ascertain the number of components, Velicer's test was utilized (36). Relationships between independent variables and outcomes in the TAM were estimated using SEM because of its appropriateness for examining theoretical frameworks, such as the TAM. SEM allows for the concurrent examination of multiple relationships between latent and observed variables (37). In the SEM, the maximum likelihood was employed as an estimator due to its suitability. To assess the model's goodness of fit, we used several indicators such as the chi-square statistic, root mean square error of approximation (RMSEA), comparative fit index (CFI), and Tucker-Lewis index (TLI). The model was considered to have an acceptable fit if it met the following benchmarks: RMSEA value of 0.08 or less, CFI of at least 0.90, and TLI of 0.90 or above. The analysis was stratified by telehealth use status (users and non-users) to explore potential differences in the factors influencing telehealth acceptance between these groups. Stratification enables a better understanding of how prior experience with telehealth may impact the relationships proposed in the TAM and helps identify tailored strategies to increase telehealth adoption. A statistical significance of *p* ≤ 0.05 was used to identify any differences and associations between variables. STATA version 18 was used for data analysis.

## 3 Results

### 3.1 Demographic characteristics

Descriptive statistics of the sample by telehealth use status are displayed in Table 1. Of the study sample (*n* = 466), 256 (55%) older adults used telehealth in the Jazan region of Saudi Arabia. A significant relationship existed between occupation status before retirement and telehealth use. Higher percentages of older adults with office-based occupations (62% vs. 38%), professional occupations (79% vs. 21%), and self-employed status (60% vs. 40%) were telehealth users. Additionally, a significant relationship was found between education level and telehealth use status, indicating that education influences telehealth adoption among older adults. Specifically, a higher percentage of illiterate older adults were telehealth non-users (55% vs. 45%), which may be attributed to lower technological literacy and discomfort with using digital platforms. However, a higher percentage of older adults with a college degree or higher were telehealth users (59% vs. 41%), possibly due to greater familiarity with technology and a better understanding of the advantages offered by telehealth services. Furthermore, a higher percentage of older adults with health insurance were telehealth users (80% vs. 20%) than those without health insurance. This indicates that health insurance may be crucial in facilitating access to telehealth services.

**Table 1:** Descriptive data about telehealth use status (row %).

**Total**		**Users**	**Non-users**	***P*-value**
		**256 (54.9%)**	**210 (45.1%)**	
Age [mean (standard deviation)]		67.9 (8.2)	68.3 (8.4)	0.687
Sex	Male	129 (58.9%)	90 (41.1%)	0.105
	Female	127 (51.4%)	120 (48.6%)	
Nationality	Saudi	249 (54.6%)	207 (45.4%)	0.333
	Non-Saudi	7 (70%)	3 (30%)	
Occupation	Office	61 (62.2%)	37 (37.8%)	< 0.001
	Professional	23 (79.3%)	6 (20.7%)	
	Self-employed	36 (60.0%)	24 (40.0%)	
	Blue-collar	22 (55.0%)	18 (45.0%)	
	Dependent	92 (43.6%)	119 (56.4%)	
	Other	22 (78.6%)	6 (21.4%)	
Education level	Illiterate	73 (45.3%)	88 (54.7%)	0.026
	≤ High school	93 (60.8%)	60 (39.2%)	
	Diploma	31 (59.6%)	21 (40.4%)	
	Bachelor or above	59 (59.0%)	41 (41.0%)	
Health insurance	Yes	76 (80.0%)	19 (20.0%)	< 0.001
	No	180 (48.5%)	191 (51.5%)	
Chronic conditions	Diabetes	76 (55.5%)	61 (44.5%)	0.369
	Hypertension	33 (60.0%)	22 (40.0%)	
	Heart disease	17 (60.7%)	11 (39.3%)	
	Gastrointestinal complications	22 (62.9%)	13 (37.1%)	
	Dyslipidemia	20 (62.5%)	12 (37.5%)	
	Arthritis	52 (48.1%)	56 (51.9%)	
	Mental illnesses	17 (42.5%)	23 (57.5%)	
	Other conditions	19 (61.3%)	12 (38.7%)	

### 3.2 Telehealth experience

Among telehealth users (*n* = 256), a high percentage (42%) reported that telehealth was better than in-person appointments. Furthermore, more than a quarter reported no difference between telehealth and traditional visits (26%). Conversely, 15% reported that telehealth was worse than traditional visits. Twelve percent of the older adults reported that they were unsure about the difference between telehealth and traditional in-person visits.

### 3.3 TAM model findings for telehealth users

The factor loading and reliability tests for all scales in this study are presented in Appendix 1. Internal reliability of the items was confirmed by values of Cronbach's alpha exceeding 0.7. SEM's goodness-of-fit measures are shown in Table 2, indicating an acceptable fit to the data. A significant chi-square result was observed [χ^2^_(124)_ = 328.836, *p* < 0.001]. Borderline acceptable fit was indicated by RMSEA value of 0.080 (90% CI: 0.070–0.091). The results of CFI (0.952) and TLI (0.941) both suggest a good to excellent fit. SEM results of telehealth users are displayed in Table 3 which indicate that PU had a significant positive effect on ATT (β = 0.501 [95% CI = 0.252–0.749]; *p* < 0.001). Facilitating conditions (β = 0.768 [95% CI = 0.324 −1.212]; p = 0.001) had a significant positive effect on ATT among telehealth users. Among telehealth users, ATT had a significant effect on BI to use telemedicine (β = 0.875 [95% CI = 0.780–0.971]; *p* < 0.001). The results suggest that ATT fully mediates the relationship between PU and BI. Specifically, PU had a significant indirect effect on BI through ATT (Î^2^ = 0.438, 95% CI [0.220, 0.656], *p* < 0.001). The total effect of PU on BI is identical to the indirect effect, indicating that PU influences BI entirely through its impact on ATT. The direct effect of PU on BI is negligible, supporting the hypothesis of full mediation. Similarly, the relationship between FC and BI is also fully mediated by ATT. FC has a significant indirect effect on BI through ATT, with the total effect of FC on BI being identical to its indirect effect.

**Table 2:** Fit statistics for structural equation model—users.

**Fit statistics**	**Value**	**Description**
**Likelihood ratio**		
chi2_ms (124)	328.836	Model vs. saturated
*p* > chi^2^	< 0.001	
chi2_bs (153)	4,423.501	Baseline vs. saturated
*p* > chi^2^	< 0.001	
**Population error**		
RMSEA	0.080	Root mean squared error of approximation
90% CI, lower bound	0.070	
Upper bound	0.091	
pclose	< 0.001	Probability RMSEA ≤ 0.05
**Baseline comparison**		
CFI	0.952	Comparative fit index
TLI	0.941	Tucker–Lewis index

**Table 3:** Structural model results: path analysis for telehealth users.

**Path**			**Standardized β**	**95% CI**	***P*-value**	**Comments**
**Direct effect**						
Perceived usefulness	→	Attitude toward use	0.501	(0.252–0.749)	< 0.001	Supported
Perceived ease of use	→	Attitude toward use	−0.196	(−0.577–0.186)	0.315	Not supported
Social impact or influence	→	Attitude toward use	0.113	(−0.03–0.256)	0.122	Not supported
Facilitating conditions	→	Attitude toward use	0.768	(0.324–1.212)	0.001	Supported
Attitude toward use	→	Behavioral intention to use	0.875	(0.78–0.971)	< 0.001	Supported
**Indirect effect**						
Perceived usefulness	→	Behavioral intention to use	0.438	(0.22–0.656)	< 0.001	Supported
Perceived ease of use	→	Behavioral intention to use	−0.171	(-0.505–0.162)	0.314	Not supported
Social impact or influence	→	Behavioral intention to use	0.099	(-0.027–0.224)	0.122	Not supported
Facilitating conditions	→	Behavioral intention to use	0.672	(0.282–1.063)	0.001	Supported
**Total effect**						
Perceived usefulness	→	Behavioral intention to use	0.438	(0.22–0.656)	< 0.001	Supported
Perceived ease of use	→	Behavioral intention to use	−0.171	(-0.505–0.162)	0.314	Not supported
Social impact or influence	→	Behavioral intention to use	0.099	(-0.027–0.224)	0.122	Not supported
Facilitating conditions	→	Behavioral intention to use	0.672	(0.282–1.063)	0.001	Supported

CI, confidence interval.

### 3.4 TAM model findings for telehealth non-users

The factor loading and reliability tests for all scales used in this study are presented in Appendix 2. The variables were internally reliable, as the Cronbach's alpha values were higher than 0.7. The goodness-of-fit measures for SEM, as presented in Table 4, indicate that the model is an overall good fit. Table 5 displays the results of the SEM model for telehealth nonusers. The results indicate that PU had a significant positive effect on ATT (β = 0.441 [95% CI = 0.230–0.651]; *p* < 0.001). Conversely, PEOU and social impact had no significant effect on ATT. Nevertheless, facilitating conditions positively affected the ATT (β = 0.807 [95% CI = 0.388 −1.226]; *p* < 0.001). Among telehealth non-users, ATT had a significant effect on the BI to use telemedicine (β = 0.902 [95% CI = 0.793–0.948]; *p* < 0.001). The results indicate that the relationship between PU and BI is fully mediated by ATT. Specifically, PU had a significant indirect effect on BI through ATT, indicating that PU influences BI entirely through its impact on ATT. The direct effect of PU on BI was negligible, supporting the hypothesis of full mediation. Similarly, the relationship between FC and BI is fully mediated by ATT. FC has a significant indirect effect on BI through ATT, with the total effect of FC on BI identical to its indirect effect. This finding indicates that FC influences BI entirely through its impact on ATT, with no meaningful direct effect, further supporting the hypothesis of full mediation.

**Table 4:** Fit statistics for SEM—non-users.

**Fit statistics**	**Value**	**Description**
**Likelihood ratio**		
chi2_ms (124)	244.858	Model vs. saturated
*p* > chi^2^	< 0.001	
chi2_bs (153)	3,813.685	Baseline vs. saturated
*p* > chi^2^	< 0.001	
**Population error**		
RMSEA	0.068	Root mean squared error of approximation
90% CI, lower bound	0.056	
Upper bound	0.081	
pclose	0.010	Probability RMSEA ≤ 0.05
**Baseline comparison**		
CFI	0.967	Comparative fit index
TLI	0.959	Tucker–Lewis index

**Table 5:** Structural model results: path analysis for telehealth non-users.

**Path**			**Standardized β**	**95% CI**	***P*-value**	**Comments**
**Direct effect**						
Perceived usefulness	→	Attitude toward use	0.441	(0.23–0.651)	< 0.001	Supported
Perceived ease of use	→	Attitude toward use	−0.260	(−0.553–0.032)	0.081	Not supported
Social impact or influence	→	Attitude toward use	−0.051	(−0.248–0.147)	0.615	Not supported
Facilitating conditions	→	Attitude toward use	0.807	(0.388–1.226)	< 0.001	Supported
Attitude toward use	→	Behavioral intention to use	0.902	(0.793–1.012)	< 0.001	Supported
**Indirect effect**						
Perceived usefulness	→	Behavioral intention to use	0.398	(0.204–0.591)	< 0.001	Supported
Perceived ease of use	→	Behavioral intention to use	−0.235	(−0.5–0.03)	0.082	Not supported
Social impact or influence	→	Behavioral intention to use	−0.046	(−0.224–0.133)	0.615	Not supported
Facilitating conditions	→	Behavioral intention to use	0.729	(0.343–1.114)	< 0.001	Supported
**Total effect**						
Perceived usefulness	→	Behavioral intention to use	0.398	(0.204–0.591)	< 0.001	Supported
Perceived ease of use	→	Behavioral intention to use	−0.235	(−0.5–0.03)	0.082	Not supported
Social impact or influence	→	Behavioral intention to use	−0.046	(−0.224–0.133)	0.615	Not supported
Facilitating conditions	→	Behavioral intention to use	0.729	(0.343–1.114)	< 0.001	Supported

CI, confidence interval.

### 3.5 Facilitators of telehealth use

**Figure 2:**
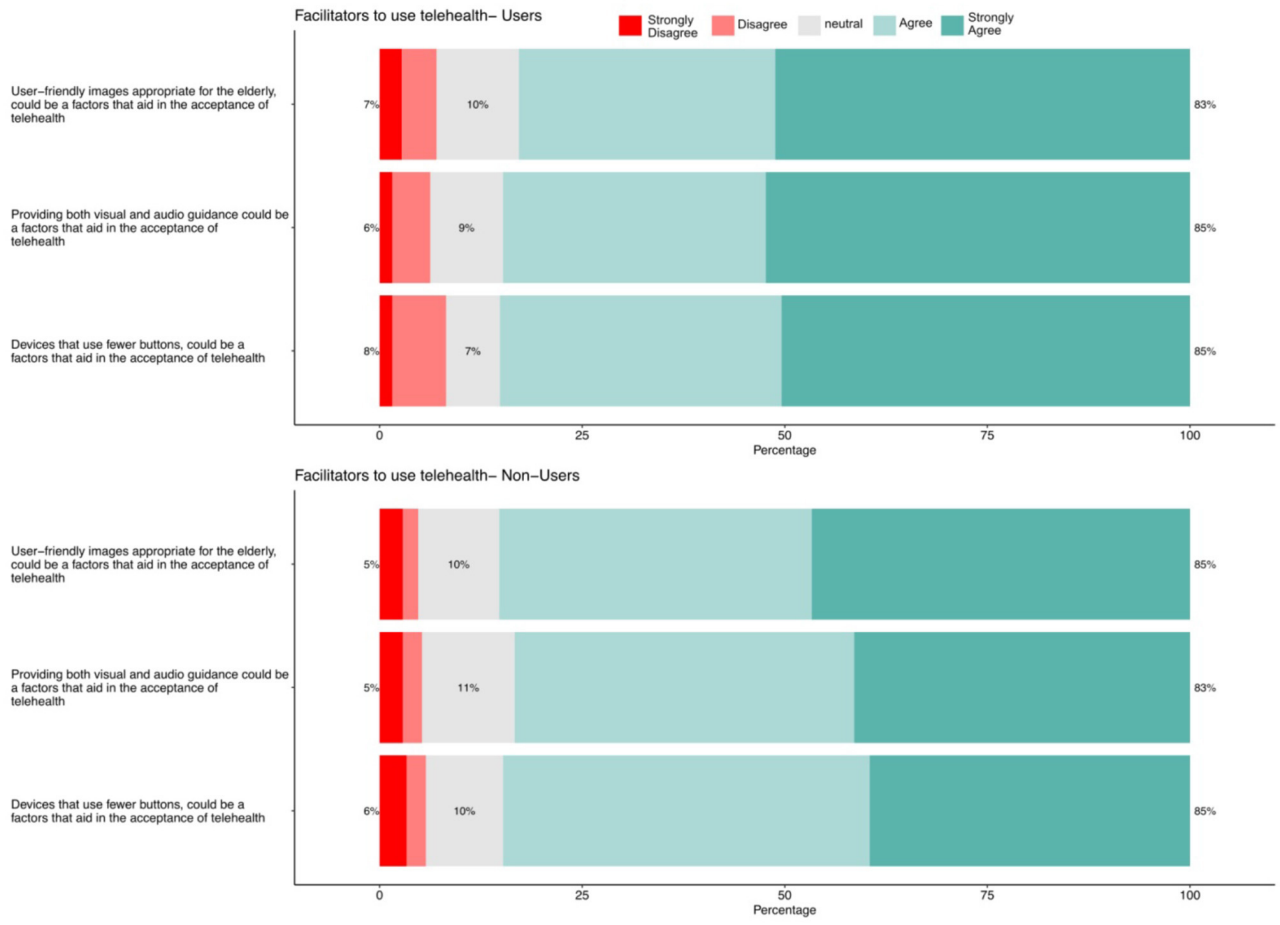
Facilitators for using telehealth.

Figure 2 shows the results regarding facilitators of telehealth use for users and non-users. Among telehealth users, a significant majority (83%) expressed either agreement or strong agreement regarding the incorporation of user-friendly images appropriate for older adults, which could enhance the acceptance of telehealth.” This suggests that visual simplicity and age-appropriate graphics are crucial in enhancing usability for older adults, who may have visual impairments or reduced familiarity with digital interfaces. Furthermore, 85% of the users agreed or strongly agreed that telehealth acceptance could be enhanced if the devices used fewer buttons and provided both audio and visual guidance. These findings indicate that simplifying device interfaces and offering multimodal guidance can significantly reduce technological barriers. For older adults, who may experience cognitive decline or physical limitations such as reduced fine motor skills, fewer buttons and clear instructions make technology more accessible and less intimidating.

Similarly, among non-users, 85% either agreed or strongly agreed with the importance of “user-friendly images” and “devices that use fewer buttons” in aiding the acceptance of telehealth. This reflects a shared recognition across both groups that simplicity and ease of use are key factors in adopting telehealth services. The high agreement among non-users highlights potential areas to focus on to increase telehealth adoption in this group.

### 3.6 Barriers to using telehealth

**Figure 3:**
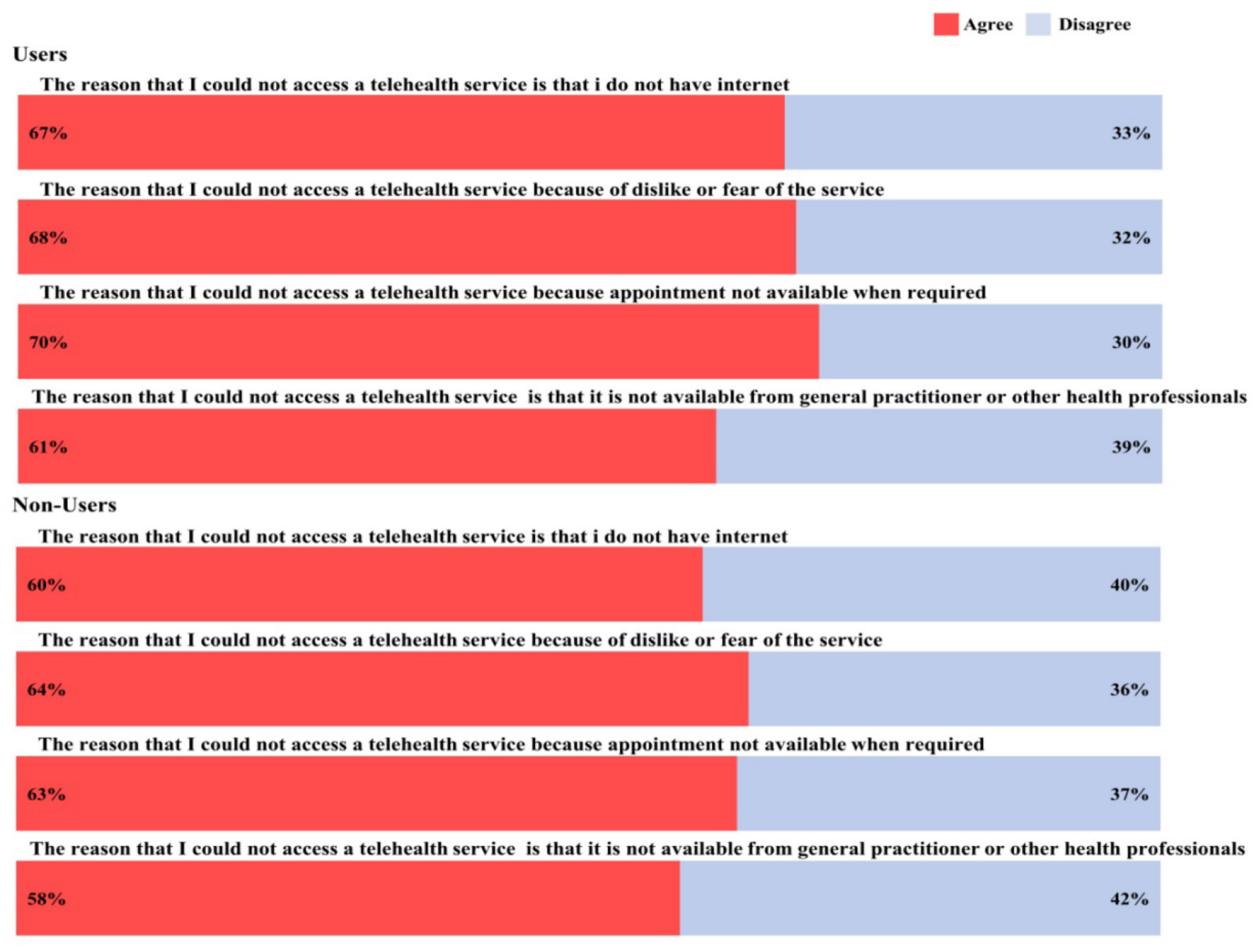
Barriers to using telehealth among users and non-users.

Figure 3 displays the results for barriers to using telehealth for users and non-users. Among telehealth users, 70% agreed that they could not access telehealth services because appointments were unavailable when required. This indicates that even those familiar with telehealth face systemic issues such as scheduling difficulties, possibly due to the limited availability of healthcare providers offering telehealth services in their area. Additionally, 68% of users agreed that “dislike or fear of the service” was a barrier. This suggests that despite using telehealth, a significant proportion of older users may still harbor apprehensions about the technology, potentially stemming from generational gaps in technology adoption or concerns about the quality of remote consultations. Furthermore, 67% of the users agreed that not having the Internet was the reason they could not access a telehealth service. This highlights the digital divide affecting even current users, where unreliable internet connectivity or lack of digital literacy can impede consistent access to telehealth services.

Among telehealth non-users, 64% agreed that “dislike or fear of the service” prevented them from accessing telehealth. This reflects a cultural preference for traditional in-person healthcare interactions and possible distrust of virtual platforms among the older adults. Similarly, 63% agreed that appointments not being available when required was a barrier, indicating that access issues are a common challenge regardless of telehealth use status.

## 4 Discussion

This study aimed to identify which key psychosocial factors best predict individuals' behavioral intention to use telehealth services, as mediated by attitude toward use, among older adults with chronic health conditions in Saudi Arabia's Jazan region and to conduct comparisons between its users and non-users. Our research findings support our hypothesis that attitude toward using telehealth would mediate the relationship between behavioral intention to use telehealth and perceived usefulness and facilitating conditions. However, our findings did not support the hypothesis that attitude toward using telehealth would mediate the relationship between behavioral intention to use telehealth and perceived ease of use and social impact. PU had a significant effect on ATT among both users and non-users. Previous studies have confirmed that PU significantly affects adoption of telehealth (38, 39). Another study reported that both PU and PEOU positively affect intention to use telehealth, with PU having a stronger influence on telehealth adoption (40).

Facilitating conditions significantly influenced the attitudes of both users and non-users toward using telehealth services. Cimperman et al., demonstrated that FC directly influenced BI of older adults to use Home Telehealth Services (41). Woo and Dowding highlighted that the availability of clinical/technical support (facilitating conditions) was associated with telehealth initiation (42). Additionally, our findings suggest that, among users and non-users, ATT significantly mediated BI to use telehealth services. This finding of positive mediation of attitude on BI is supported by another study in which participants had a positive attitude toward using telehealth and was correlated with the BI toward use of telehealth in the future (43). Kohnke et al. included attitude as a moderator in their structural equation modeling (SEM) for predicting BI to use telehealth (44).

The findings of this research show that PU has a significantly beneficial impact on the BI (mediated by ATT) to use telehealth among the Saudi older population. Comparable results were found by Cimperman et al., where performance expectancy (which is closely related to PU) had a direct impact on the BI to use home telehealth services (41). Another Chinese study on the adoption of telehealth indicated that PU significantly and positively affected BI among older adults (45). However, a study from the Philippines reported that effort expectancy has a more significant influence on BI to use telehealth than other factors (46). Previous studies have also identified other factors, such as social impact (47) and system self-efficacy (48), which may have considerable influence on the adoption of telehealth.

Our findings underscore the importance of telehealth services being perceived as useful in managing health conditions, especially among those already using telehealth. Thus, enhancing the PU of telehealth services is essential for widespread acceptance. Intuitive design and clear demonstration of benefits can influence perceptions about the usefulness of telehealth systems. The practical implications of these findings suggest that healthcare providers should actively promote the benefits of telehealth to older patients, highlighting how it can improve health management and access to care. Providers can address training needs by offering practical demonstrations, informational materials, and personalized support to increase patients' familiarity and comfort with telehealth technologies. It is suggested that technology designers integrate user-friendly interfaces tailored to the capabilities of older adults. Simplifying technology interfaces using larger fonts, clear icons, and straightforward navigation, as well as providing visual and audio guidance, can make telehealth platforms more accessible. Improvements should be implemented in telehealth-related technologies, such as bio-physiological data monitoring tools, integration of data with electronic medical records, and connection of data with multiple sources to enhance functionality and appeal to users and potential users.

Policymakers have a crucial role in addressing barriers to telehealth adoption among older adults. Although Saudi Arabia has made significant strides in expanding internet infrastructure as part of Vision 2030—achieving 100% mobile-cellular network coverage and high percentages for 3G and 4G coverage even in rural areas (49)—the high percentage of participants citing lack of internet access as a barrier suggests that challenges remain. These challenges may not stem solely from infrastructural deficiencies but could include factors such as the affordability of internet services, lack of access to suitable devices, or digital literacy gaps among the older population. Therefore, policymakers should not just focus on maintaining and enhancing infrastructure but also work toward affordability and accessibility of internet services and devices to the older adults. The provision of training programs for older adults are an important initiative that can improve digital literacy. These initiatives are in line with the country's long-term plan (Vision 2030), which aims to achieve digital transformation, inclusivity, and transformation of the healthcare sector. Implementation of these strategies could substantially enhance the adoption of telehealth among older adults and ensure the inclusion of all segments of society.

The research was conducted in Saudi Arabia's Jazan region, the country's second-smallest region, with 40% of its population living in rural settings (50). The results reveal that technological obstacles, such as insufficient internet connectivity, and personal factors, including fear or dislike of telehealth services, significantly affect older adults' use of telehealth. The high proportion of poor internet access (67% of users and 60% of non-users) highlights the digital gap in rural areas, where challenging terrain and scattered populations limit the technological infrastructure. Cultural elements, such as resistance to change and preference for in-person interactions (68% of users and 64% of non-users expressing fear or dislike of telehealth services), exacerbate these challenges. The older generation, which has grown without modern technology, is less comfortable with complex digital platforms and finds it challenging to navigate.

From a demographic perspective, older adults frequently encounter difficulties such as declining eyesight, hearing loss, and decline in motor skills, making user-friendly designs critical for the adoption of telehealth systems. This is underscored by 83%−85% of both users and non-users expressing their agreement on preferring easy-to-understand visuals and devices with minimal buttons on telehealth platforms. Additionally, telehealth interfaces should be simplified and include audio and visual support to enhance usability and acceptance among older adults. Focused public health awareness campaigns should be undertaken to educate older adults regarding the benefits of telehealth and address their fears regarding its use. To address these challenges and capitalize on facilitators, a holistic and culturally aware strategy tailored to specific demographic requirements is necessary, which should aim to improve the technological infrastructure, boost digital literacy, and build confidence in older adults with regard to the use of telehealth services. Steps should be taken to improve internet access in rural areas, and community programs should be implemented to train older adults to use digital devices and improve their digital literacy. Health technology companies should design customized interfaces based on the preferences of older adults, obtain extensive feedback from users, and undergo evaluation by healthcare providers to ensure mapping of the needs and telehealth solutions. Health authorities should provide training programs to health care professionals on the use of telehealth services and strategies to provide support to older adults. Special training should be provided to health care professionals when dealing with older adults with visual or hearing disabilities. From the healthcare providers' perspective, identifying the training needs is crucial to understanding the gaps and providing hands-on demonstrations to the older adults to build their confidence and mitigate their apprehensions associated with the use of telehealth.

A potential limitation of this study is that it was conducted only in the Jazan region, which may limit the generalizability of the findings to other regions of Saudi Arabia. The cultural, infrastructural, and socioeconomic characteristics of Jazan may differ from those of other areas, impacting telehealth acceptance differently. Additionally, the use of snowball sampling might introduce selection bias, as participants may refer others with similar characteristics or ATT. This method may not capture a fully representative sample of the older population. Our study lacked data on the frequency of telehealth utilization among participants, preventing us from distinguishing between one-time users and those who had multiple or ongoing sessions, which may vary considerably. Future investigations should include this variable to offer a more detailed understanding of telehealth experiences at different usage levels. Another limitation of our study is the analysis of facilitators and barriers to the use of telehealth services. We employed a descriptive approach that restricted the deeper insights provided by the inclusion of a qualitative approach. Participants were asked to select only from the options provided instead of expressing their own views through open-ended questions. Employing a mixed-methods approach combining both qualitative and quantitative techniques could benefit future studies by gaining a more comprehensive understanding of telehealth usage in lieu of facilitators and barriers. An additional limitation was the potential lack of clarity of certain survey items, as they were not tailored to capture specific information. Our questionnaire was aimed at gathering broader perceptions and attitudes toward the use of telehealth, permitting a broad understanding of the factors that influence the adoption and use of telehealth.

The Technology Acceptance Model (TAM) was applied as the conceptual model for this study as it has been widely applied across various information technology domains, which demonstrates its generalizability to some extent. Nevertheless, the generalizability of the TAM is not without limitations or contradictions. Some studies have found inconsistencies in the TAM's applicability, especially when applied to specific user groups or technologies. For example, in the healthcare sector, perceived ease of use did not predict intention to use online health applications by physicians (51). Similarly, regarding electronic collaboration technology, perceived usefulness was found to have a negative association with system usage, thereby contradicting the typical findings of the TAM (52). In view of such inconsistencies, the generalizability of TAM may vary depending on the specific technology and the user group under study, and caution should be exercised when extrapolating our findings to a larger scale.

To improve the applicability of findings on a larger scale, future studies should be conducted across various regions in Saudi Arabia. This approach would allow for a comparison of results and to identify the regional differences in the acceptance of telehealth services. Expanding the research to include younger populations or different demographic groups could provide comparative insights into telehealth acceptance across age groups. Additionally, longitudinal studies examining changes in attitudes and use over time would be valuable, especially as technology evolves and becomes more integrated into healthcare services. Investigating other adoption models beyond the TAM could also offer a deeper understanding of factors influencing telehealth acceptance. This study has significance for guiding future research, as well as for Saudi Arabian healthcare policymakers.

## 5 Conclusion

This study examined the factors influencing ATT and BI to use telehealth among older adults with chronic conditions in the Jazan region of Saudi Arabia. Key findings indicate that PU and facilitating conditions positively affect ATT among both users and non-users. Barriers such as lack of internet access and fear or dislike of telehealth services hinder use, whereas facilitators such as user-friendly interfaces and simplified devices enhance acceptance. These findings have important practical implications. For healthcare providers, a need exists to address training and support for older patients by offering education on how to use telehealth technologies and emphasizing their benefits. Policymakers should focus on improving internet infrastructure in rural areas, subsidizing internet access and devices for older adults, and developing policies encouraging telehealth adoption. Telehealth application developers should design interfaces that meet the needs of older adults users, incorporating simplified navigation, clear visual aids, and multimodal guidance to enhance usability.

Altogether, this study advances the understanding of telehealth by highlighting the key factors that influence the adoption of telehealth among older adults in Saudi Arabia. The study findings can aid healthcare providers and policymakers in understanding the barriers in existing support systems and taking steps to address them. They can also use facilitators to improve the accessibility of healthcare technology, promote independence, and enhance the quality of life of older adults through tailored telehealth educational programs, thereby leading to better health outcomes and supporting the healthcare transformation of Saudi Arabia.

## Data Availability

The raw data supporting the conclusions of this article will be made available by the authors, without undue reservation.
