# Supplementary material for: Telehealth perceptions and associated factors among older adults with chronic conditions in Saudi Arabia: a comparative study of users and non-users
Source: Front Public Health. 2025 Mar 12;13:1542974. doi: 10.3389/fpubh.2025.1542974 (PMC11936876; doi:10.3389/fpubh.2025.1542974)
Supplement: Supplementary file 1 [file Table_1.docx]

Appendix-1: Users

| Scale | Item | Single Factor Loadings | Cronbach’s Alpha |
| --- | --- | --- | --- |
| Perceived Usefulness | Health Care using telehealth will help me manage my health | 0.730 | 0.88 |
|  | I believe that using telehealth will make my daily life safer | 0.910 |  |
|  | Using telehealth will improve my quality of life | 0. 897 |  |
| Perceived Ease of Use | I think using telehealth would be simple | 0.836 | 0.88 |
|  | Learning to use telehealth application will be easy | 0.846 |  |
|  | Telehealth-Health care will be convenient to use | 0.840 |  |
| Social impact or Influence | Family will approve of my use of telehealth | 0.760 | 0.89 |
|  | Acquaintances will recommend that I use telehealth | 0.947 |  |
|  | Acquaintances will approve of me using telehealth | 0.889 |  |
| Facilitating conditions | I will know how to use telehealth | 0.820 | 0.82 |
|  | If I encounter difficulties using your telehealth, I think someone will be able to help | 0.768 |  |
|  | I have sufficient resources to use telehealth | 0.760 |  |
| Attitude towards use | Using telehealth will have a positive impact on my life | 0.902 | 0.89 |
|  | Using a telehealth will benefit my health | 0.893 |  |
|  | I have positive thoughts about telehealth | 0.750 |  |
| Behavioral Intention to Use | I would use telehealth if given the opportunity | 0.852 | 0.91 |
|  | I will use telehealth for my health care | 0.908 |  |
|  | I will use telehealth change my life for the better | 0.892 |  |
