# Supplementary material for: Telehealth perceptions and associated factors among older adults with chronic conditions in Saudi Arabia: a comparative study of users and non-users
Source: Front Public Health. 2025 Mar 12;13:1542974. doi: 10.3389/fpubh.2025.1542974 (PMC11936876; doi:10.3389/fpubh.2025.1542974)
Supplement: Supplementary file 2 [file Table_2.docx]

Appendix-2: Non-users

| Scale | Item | Single Factor Loadings | Cronbach’s Alpha |
| --- | --- | --- | --- |
| Perceived Usefulness | Health Care using telehealth will help me manage my health | 0.789 | 0.90 |
|  | I believe that using telehealth will make my daily life safer | 0.909 |  |
|  | Using telehealth will improve my quality of life | 0.896 |  |
| Perceived Ease of Use | I think using telehealth would be simple | 0.843 | 0.90 |
|  | Learning to use telehealth application will be easy | 0.906 |  |
|  | Telehealth-Health care will be convenient to use | 0.859 |  |
| Social impact or Influence | Family will approve of my use of telehealth | 0.837 | 0.91 |
|  | Acquaintances will recommend that I use telehealth | 0.926 |  |
|  | Acquaintances will approve of me using telehealth | 0.892 |  |
| Facilitating conditions | I will know how to use telehealth | 0.835 | 0.83 |
|  | If I encounter difficulties using your telehealth, I think someone will be able to help | 0.812 |  |
|  | I have sufficient resources to use telehealth | 0.730 |  |
| Attitude towards use | Using telehealth will have a positive impact on my life | 0.931 | 0.90 |
|  | Using a telehealth will benefit my health | 0.923 |  |
|  | I have positive thoughts about telehealth | 0.772 |  |
| Behavioral Intention to Use | I would use telehealth if given the opportunity | 0.899 | 0.94 |
|  | I will use telehealth for my health care | 0.974 |  |
|  | I will use telehealth change my life for the better | 0.900 |  |
